# Assembly-Induced Emission of Copper Nanoclusters: Revealing the Sensing Mechanism for Detection of Volatile Basic Nitrogen in Seafood Freshness On-Site Monitoring

**DOI:** 10.1021/acsami.3c13321

**Published:** 2024-01-23

**Authors:** Chenyue Zhou, Da-Wen Sun, Ji Ma, Anjun Qin, Ben Zhong Tang, Xiao-Ru Lin, Shi-Lin Cao

**Affiliations:** † School of Food Science and Engineering, South China University of Technology, Guangzhou 510641, China; ‡ Academy of Contemporary Food Engineering, South China University of Technology, Guangzhou Higher Education Mega Centre, Guangzhou 510006, China; § Engineering and Technological Research Centre of Guangdong Province on Intelligent Sensing and Process Control of Cold Chain Foods, & Guangdong Province Engineering Laboratory for Intelligent Cold Chain Logistics Equipment for Agricultural Products, Guangzhou Higher Education Mega Centre, Guangzhou 510006, China; ∥ Food Refrigeration and Computerized Food Technology (FRCFT), Agriculture and Food Science Centre, University College Dublin, National University of Ireland, Belfield, Dublin 4, Ireland; ⊥ State Key Laboratory of Luminescent Materials and Devices, Center for Aggregation-Induced Emission, South China University of Technology, Guangzhou 510640, China; # Shenzhen Institute of Aggregate Science and Technology, School of Science and Engineering, The Chinese University of Hong Kong, Shenzhen 518172, China; ▽ Guangdong Key Laboratory of Food Intelligent Manufacturing, Foshan University, Foshan 528000, China

**Keywords:** aggregation-induced emission, copper nanocluster, self-assembly, seafood freshness, sensing

## Abstract

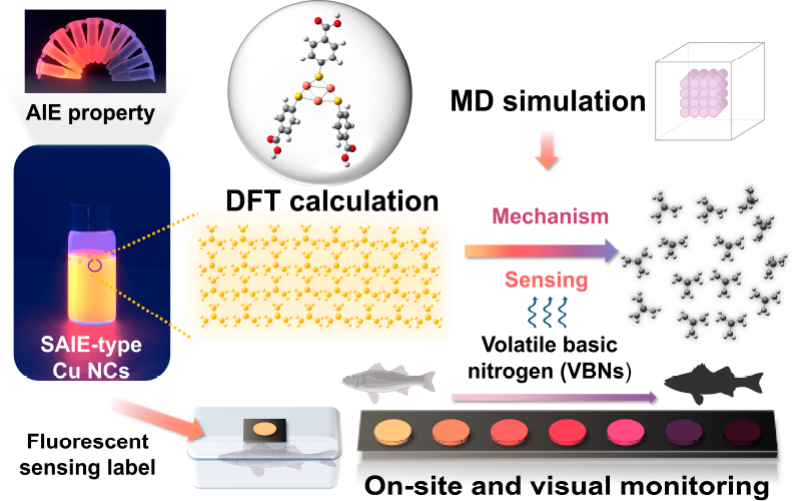

Total volatile basic nitrogen (TVB-N) is a vital indicator for assessing seafood freshness and edibility. Rapid on-site detection of volatile basic nitrogen (VBN) is of significant importance for food safety monitoring. In this study, highly luminescent self-assembled copper nanoclusters (Cu NCs@*p*-MBA), synthesized using *p*-mercaptobenzoic acid (*p*-MBA) as the ligand, were utilized for the sensitive detection of VBNs. Under acidic conditions, Cu NCs@*p*-MBA formed compact and well-organized nanosheets through noncovalent interactions, accompanied by intense orange fluorescence emission (651 nm). The benzene carboxylic acid part of Cu NCs@*p*-MBA provided the driving force for supramolecular assembly and exhibited a strong affinity for amines, particularly low-molecular-weight amines such as ammonia (NH_3_) and trimethylamine (TMA). The quantitative determination of NH_3_ and TMA showed the detection limits as low as 0.33 and 0.81 ppm, respectively. Cu NCs@*p*-MBA also demonstrated good responsiveness to putrescine and histamine. Through density functional theory (DFT) calculations and molecular dynamics (MD) simulations, the precise atomic structure, assembly structure, luminescent properties, and reaction processes of Cu NCs@*p*-MBA were studied, revealing the sensing mechanism of Cu NCs@*p*-MBA for highly sensitive detection of VBNs. Based on the self-assembled Cu NCs@*p*-MBA nanosheets, portable fluorescent labels were developed for semiquantitative, visual, and real-time monitoring of seafood freshness. Therefore, this study exemplified the high sensitivity of self-assembly induced emission (SAIE)-type Cu NCs@*p*-MBA for VBNs sensing, offering an efficient solution for on-site monitoring of seafood freshness.

## 1 Introduction

Volatile amines and biogenic amines (BAs) are widely generated by the degradation of amino acids in biological metabolism.^1^ High levels of these compounds can serve as indicators for various diseases and may act as potential references for the deterioration of high-protein foods.^2^ Total volatile basic nitrogen (TVB-N), including biogenic amines and ammonia, has been recognized as an important indicator for evaluating seafood freshness and edibility.^3^ Conventional techniques for detecting volatile basic nitrogen (VBNs) rely on gas chromatography, liquid chromatography, capillary electrophoresis, and semimicro-Kjeldahl nitrogen determination. However, these analytical methods normally require lengthy detection, tedious sample pretreatment, expensive instruments, and skilled technicians.^4^ In contrast, fluorescent sensors are more user-friendly due to their rapid response, simple operation, high sensitivity, and cost-effective measurements.^5^ In the exploration for fluorescent sensors, aggregation-induced emission (AIE) fluorescent materials have become a focal point in the sensing field, due to their inherent capability in constructing solid-state sensors, and show great potential in nondestructive and visual monitoring of seafood spoilage.^6^ The advantage of the AIE principle lies in designing molecules with highly twisted structures to ensure high emission efficiency in the solid state. This avoids the aggregation-induced quenching (ACQ) effect caused by intermolecular π–π stacking and excimer formation. The AIE concept has seen rapid expansion, starting from the discovery of the initial organic molecule, hexaphenylsilole (HPS), to diverse domains, encompassing nanodots, metal nanoparticles, and metal nanoclusters.^7^

Over the years, there has been a growing interest in using the aggregation-induced emission (AIE) strategy to acquire highly luminescent metal nanoclusters.^8^ Noble metal nanoclusters (NCs), such as gold, silver, and copper NCs, generally comprise a few to hundred metal atoms with a metallic core protected by a monolayer of organic ligands.^9^ They range in size from a few tenths to 2 nm and serve as bridges between atoms and nanocrystals.^10^ The remarkable quantum confinement effect enables metal NCs to exhibit unique molecular-like properties, such as discrete electronic transitions, size-dependent fluorescence emission, molecular chirality, intrinsic magnetism, catalytic properties, and photoluminescence.^11^ Compared to organic AIE molecules and traditional quantum dots, noble metal NCs are free from toxic heavy metals, exhibiting improved biocompatibility and low toxicity in practical applications. However, the photoluminescence (PL) of metal NCs is not competitive, with their quantum yields (QY) rarely exceeding 0.1%.^12^ Especially, the emission of individual Cu NCs is less weak in contrast to Ag and Au NCs.^13^ Developing aggregated or assembled structures using the AIE design principles has facilitated the synthesis of highly luminescent metal NCs, enhancing QY to the range of 5–20%.^12^ Notably, small-sized metal NCs often suffer from irregular and unstable aggregates due to their high surface energy, resulting in poor color purity and fluorescence quenching.^14^ Therefore, compared to typical AIE systems, the self-assembly induced emission (SAIE) strategy is conducive to constructing metal NCs with better performances and high photoluminescence quantum yields (PLQYs).^15^

The design of solid-state AIE sensors using self-assembled molecules provides a bottom-up approach for constructing sensitive, fast, adaptable, and reversible sensing systems. For example, Han et al. used three AIE positional isomers (DB) to fabricate the customized AIE sensors, achieving ammonia (NH_3_) detection with a LOD of 2.02 Pa.^16^ The three isomers self-assembled into different morphologies, including 1D nanowire, 2D microsheet, and 3D microcube, and showed fluorescence quenching responses toward gaseous NH_3_. By employing density functional theory (DFT), they delved into the sensing mechanism, revealing that the interaction of ammonia molecules with the isomers led to the attenuation of the intramolecular charge transfer (ICT) effect. However, few reports are available on the design of functional materials by self-assembly of metal NCs.^1^ The development of SAIE-type metal NCs is primarily hindered by the following limitations: (1) a lack of precise knowledge about the atomic structure and photophysical fundamentals of luminescent metal NCs, especially at the molecular level; (2) limited exploration and theoretical validation of the sensing mechanisms of analytes, primarily due to the inherent challenges in obtaining unambiguous information on the complex bonding details of the high-dimensional structure. The insufficient understanding of the structures and mechanisms has impeded the further development of customizable and highly sensitive sensing platforms based on metal NCs.^12^ Consequently, to guide the construction of fluorescent sensors for broader applications in detecting analytes, it is necessary to further explore the correlation between the assembly morphology, functionality, and building blocks of metal NCs based on the atomically precise structure.^17^

Therefore, in the current study, an atomically precise SAIE-type Cu NC [Cu_3_(H_2_mba)_3_] (H_2_mba = *p*-mercaptobenzoic acid) was reported rapid detection of volatile basic nitrogen (VBNs) through fluorescence response. In a one-pot method, the ligand *p*-mercaptobenzoic acid (*p*-MBA), containing thiol groups, acted as a reducing agent and effectively coordinated with copper, resulting in the successful synthesis of Cu NCs@*p*-MBA. Owing to hydrogen bonding interactions within the carboxylic acid groups of *p*-MBA and π–π stacking facilitated between the rigid benzene rings, Cu NCs@*p*-MBA exhibited an organized nanosheet assembly structure and emitted bright orange fluorescence. Through density functional theory (DFT) calculations, the ultrasmall structure of Cu NCs@*p*-MBA was explored, and molecular dynamics (MD) simulations were employed to investigate the assembly structure, luminescent properties, and reaction processes of Cu NCs@*p*-MBA, revealing the sensing mechanism of the high sensitivity of Cu NCs@*p*-MBA in detecting VBNs. The compact nanosheet assembly structure of Cu NCs@*p*-MBA facilitated the efficient capture of low-molecular-weight VBNs, notably ammonia and trimethylamine, the main contributors to TVB-N. Consequently, the prepared fluorescent labels were suitable for semiquantitative, in situ, and real-time monitoring of the freshness status of seafood (sea bass). Notably, this study represents the first example where SAIE-type metal NCs could be effectively used to detect volatile amines for in situ monitoring of food freshness.

## 2 Materials and Methods

### 2.1 Materials, Reagents, and Apparatus

Copper nitrate (Cu(NO_3_)_2_·3H_2_O, 99.0%), *p*-mercaptobenzoic acid (*p*-MBA, 95%), tetrahydrofuran (THF), *N*,*N*-dimethylformamide (DMF), trimethylamine (TMA), and dimethylamine (DMA) were purchased from Macklin Biochemical Co., Ltd. (Shanghai, China). Nitric acid (HNO_3_), ammonium hydroxide (NH_3_·H_2_O), sodium hydroxide (NaOH), ethanedioic acid (oxalic acid), dichloromethane (CH_2_Cl_2_), ethanol (EtOH), methanol (MeOH), dimethyl sulfoxide (DMSO), ethyl acetate (EtOAc), and isopropanol (IPA) were supplied by Aladdin Bio-Chemical Technology Co., Ltd. (Shanghai, China). Histamine, cadaverine, spermine, spermidine, tyramine, and putrescine were obtained from Sigma-Aldrich Co., Ltd. (St. Louis, MO). All chemicals were of analytical grade and used without further purification. Fresh sea bass fish (deceased) were bought from a local market (Guangzhou, China). The transparent containers (500 and 5000 mL) for preserving fish samples were purchased from Anbao Packing Co., Ltd. (Chengdu, China). The fiber cotton for preparing the fluorescent labels was bought from Unicharm Co., Ltd. (Tokyo, Japan). Throughout the experiments, ultrapure water (18.2 MΩ cm) purified by a Milli-Q system (Millipore Co., Billerica, MA) was used to prepare solutions. Prior to experiments, all glassware was carefully cleaned with newly made aqua regia, rinsed with Mill-Q water, and dried in a blast drying oven (DHG-9015A, Shanghai Yiheng Scientific Instruments Co., Ltd., Shanghai, China).

The ultraviolet–visible (UV–vis) absorption spectra from 200 to 700 nm were obtained using a UV-1800 spectrophotometer (Shimadzu Ltd., Kyoto, Japan). Fluorescence spectroscopy was recorded by an RF-6000 fluorescence spectrophotometer (Shimadzu Ltd., Kyoto, Japan) at an excitation wavelength of 342 nm. Under a 365 nm UV lamp (SJZX-SZX, Shengli Photonics Technology Co., Ltd., Zhongshan, China), all fluorescent photos were captured using a smartphone (Apple Inc., Cupertino, CA). A Nicolet-iS50 infrared spectrometer (Thermo Fisher Scientific Inc., Waltham, MA) was used to collect Fourier-transform infrared spectra (FT-IR). The binding energy spectra of Cu NCs@*p*-MBA were recorded by a K-Alpha X-ray photoelectron spectrometer (XPS) system (Thermo Fisher Scientific Inc.). The Raman spectra were taken by a laser confocal microscope Raman spectrometer (LabRAM HR, Horiba France SAS, Villeneuve d’Ascq, France) equipped with a 785 nm laser. Negative-ion electrospray ionization mass spectrometry (ESI-MS) measurements were recorded by an Agilent 1290/Bruker maXis impact mass spectrometer (Bruker Inc., Billerica, MA), equipped with a conventional ESI source. A STA2500 thermal gravimetric analyzer (Netzsch Co., Ltd., Selb, Germany) was used to perform thermogravimetric analysis (TGA) in an N_2_ atmosphere from 40 to 900 °C at 10 °C min^–1^. Low-field nuclear magnetic resonance (LF-NMR) equipment (NM42-SCUTH-I, Niumag Co., Ltd., Shanghai, China) was used to determine the transverse relaxation time (*T*_2_) of the self-assembled Cu NCs@*p*-MBA aggregates. Dynamic light scattering (DLS) and zeta potential were tested by a Zetasizer Nano potentiometric analyzer (Malvern Instruments Ltd., Melvin, UK). The morphology and structure images of the synthesized Cu NCs@*p*-MBA were obtained with a Talos F200X G2 transmission electron microscope (TEM) (Thermo Fisher Scientific Inc.). Scanning electron microscopic (SEM) imaging of Cu NCs@*p*-MBA and energy-dispersive X-ray analysis (EDX) mapping were conducted with a JSM-7610F scanning electron microscope (JEOL Ltd., Tokyo, Japan). The length and width of the nanosheets of the self-assembled Cu NCs@*p*-MBA in the SEM images were analyzed using ImageJ software (Version 1.53a, National Institutes of Health, New York).^18^ The microstructure and nanosheet thickness of the self-assembled Cu NCs@*p*-MBA were investigated using atomic force microscopy (AFM, SmartSPM-1000, HORIBA France SAS, Lille, France). Small-angle X-ray diffraction (SXRD) patterns for the self-assembled Cu NCs@*p*-MBA were obtained by using an Empyrean X-ray Diffractometer (Malvern Panalytical Ltd., Almelo, Netherlands), where Cu K radiation (λ = 1.54 Å) was employed. The absolute photoluminescence (PL) quantum yield (QY) of the self-assembled Cu NCs@*p*-MBA was measured using an absolute QY spectrometer C9920-02 (Hamamatsu Ltd., Tokyo, Japan) with an integrating sphere. An FLS1000 spectrometer (Edinburgh Instrument Ltd., Livingston, UK) was employed to measure the fluorescence lifetime of Cu NCs@*p*-MBA.

### 2.2 Synthesis of Cu NCs@*p*-MBA

For the synthesis of Cu NCs@*p*-MBA, a 0.0463 g amount of *p*-MBA (0.1 M) was first dissolved in 3 mL of ultrapure water, followed by adding 4 mL of THF. Under vortexing using a vortex mixer (NP-30s, Toman Instrument Co., Ltd., Changzhou, China), the mixture turned clear. Then, Cu(NO_3_)_2_ (0.1 M, 3 mL) in nitric acid (0.1 M) was added to the clear solution, resulting in precipitation. The above mixture was incubated in the dark at a 70 °C water bath (HH-1, Guohua Electric Co., Ltd., Changzhou, China) for 0.5 h. The obtained yellow solution was naturally cooled to room temperature (25 °C) and then washed thoroughly with ethanol three times to remove excess ligands. The purified product was resuspended into ethanol to be utilized for subsequent purposes.

### 2.3 Density Functional Theory (DFT) Calculations

The first-principle calculation for Cu NCs@*p*-MBA was based on the density functional theory (DFT) method with the Gaussian 16 program package (version B.01, Gaussian, Inc., Wallingford, CT).^19^ At the hybrid (B3LYP) level, the geometric structures of Cu NCs@*p*-MBA were optimized with the basis sets 6-311++G for the C, O, H, and S atoms and LANL2DZ for the Cu atom. In the case of using a double-ξ basis set, given that molecular passivation may result in the formation of unusual bonds, d polarization functions were added for S and C atoms, and p polarization functions were added for H atoms. To eliminate imaginary frequencies and ensure the accuracy of the findings, vibration frequency calculations were conducted on all obtained structures using the same analytical approach. The simulated UV–vis absorption spectrum of Cu NCs@*p*-MBA was studied based on the time-dependent DFT (TD-DFT). By using the Multiwfn 3.8 program (Beijing Kein Research Center for Natural Sciences, Beijing, China), electrostatic potential (ESP) analysis was performed.^20^

### 2.4 Molecular Dynamics (MD) Simulations

To investigate the dynamic behavior and structural changes of SAIE-type Cu NCs@*p*-MBA during fluorescence quenching (ammonia response and alkaline environment), molecular dynamics (MD) simulations were carried out using Gromacs 2019.1 software package (GROMACS Development Team).^21^ The topology files of Cu NCs@*p*-MBA and Cu NCs@*p*-MBA-DP (deprotonated) were generated using Sobtop Tool (Version 1.0, Beijing Kein Research Center for Natural Sciences, Beijing, China, http://sobereva.com/soft/Sobtop). The ACPYPE service (European Bioinformatics Institute, Cambridge, UK) was employed to generate force field files for NH_3_ and NH_4_^+^.^22^ Cu NCs@*p*-MBA nanosheets with a uniform size of 2.25 nm were subjected to a 7 × 7 × 7 nm simulation box. The AIE MD simulations in an aqueous system, designated as SOL-AIE, were performed as controls using the AMBER force field parameters.^23^ Four simulation models were constructed: (1) self-assembled Cu NCs@*p*-MBA nanosheets, (2) deprotonated Cu NCs@*p*-MBA, (3) Cu NCs@*p*-MBA in contact with NH_3_, and (4) CuNCs@*p*-MBA after reacting with NH_3_. In each model, an appropriate number of Na^+^ ions were added to maintain electrical neutrality within the simulation system. The simulation system was subjected to energy minimization at 298.15 K for 50 000 steps using the particle-mesh Ewald method.^24^ Subsequently, position-constrained MD simulations were carried out to equilibrate the AIE molecules and surrounding molecules under the NVT ensemble under the condition of constant number of particles (*N*), volume (*V*), and temperature (*T*) with periodic boundary conditions in *x*, *y*, and *z* directions, followed by NPT ensemble simulations with constant number of particles (*N*), pressure (*P*), and temperature (*T*). The Gromacs 2019.1 software package was employed to analyze hydrogen bonds (H-Bond), density, Coulomb forces (Coul-SR), and Lennard–Jones potential energy (LJ-SR) in each model.

### 2.5 Sensitivity and Selectivity of Cu NCs@*p*-MBA Indicators

Volatile basic nitrogen (VBNs), including ammonia, histamine, cadaverine, putrescine, spermine, spermidine, and tyramine, were chosen to indicate the sensitivity of the self-assembled Cu NCs@*p*-MBA. Different kinds of biogenic amines (500 μL, 50 ppm) were mixed with 500 μL of the self-assembled Cu NCs@*p*-MBA aggregates (2.5 mM) for 3 min, and the fluorescence spectra were subsequently recorded. The self-assembled Cu NCs@*p*-MBA (5 mM, in ethanol) were fully dispersed on the neatly cut fiber cotton discs with a diameter of 1 cm. Subsequently, the soaked fluorescent labels were evaporated for 20 min. The prepared labels, loaded with self-assembled nanosheets, were exposed to amine vapors and some interfering substances, including MeOH, EtOH, IPA, water, H_2_S, and EtOAc, to evaluate the specificity of the labels.

### 2.6 Cu NCs@*p*-MBA-Based Fluorescent Labels in Seafood Spoilage Monitoring

Fresh sea bass (*Lateolabrax Japonicus*) (deceased) were purchased from a local supermarket (Guangzhou, China) and then sliced into uniform fillets, each weighing 40 g. Subsequently, each fillet (40 g) was individually placed within a 500 mL transparent container. The fish samples were divided into two parallel groups, with one group undergoing storage at 4 °C for 8 days in a refrigerator (BCD-539 wt, Haier Group Co., Ltd., Shandong, China) and the other at 25 °C for 18 h using a constant temperature incubator (BPH-9402, Shanghai Yiheng Scientific Instruments Co., Ltd., Shanghai, China). The fluorescent labels were preadhered to the lid to receive VBNs during the fish spoilage. In storage at 4 and 25 °C, the fluorescent labels were photographed every 24 and 1 h, respectively, under the UV lamp (SJZX-SZX, Shengli Photonics Technology Co., Ltd., Zhongshan, China) to record the change in color. As a reference, the TVB-N content in fish fillets was measured following European Commission regulations (EC No. 1022/2008) by a standard method (Kjeldahl distillation) to determine the freshness levels of fish samples. The TVB-N limit values for determining the spoilage and severe decay of fish fillets were 25 and 30 mg/100 g.^25^

To further assess the accuracy of the sensing labels, whole fresh sea bass (deceased) samples weighing approximately 400 to 500 g each were individually placed in 5000 mL transparent containers. These containers were equipped with preattached fluorescent labels designed to capture VBNs. The fish samples were divided into two groups, with one group stored at 4 °C and the other at 25 °C, to conduct freshness monitoring experiments. Under these conditions (4 and 25 °C), fluorescent labels were photographed every 48 and 3 h, respectively, to document color changes. The color of the labels was then compared to the designed color card to assess the freshness of the fish. Simultaneously, the TVB-N content of the fish was measured to validate the accuracy of the labels.

### 2.7 Statistical Analysis

All experiments were carried out in triplicate, and the outcomes were presented as the mean value ± standard deviation. The collected data were processed using OriginPro 9.8 software (OriginLab Co., Northampton, MA).

## 3 Results and Discussion

### 3.1 Characterization of Cu NCs@*p*-MBA

**Figure 1:**
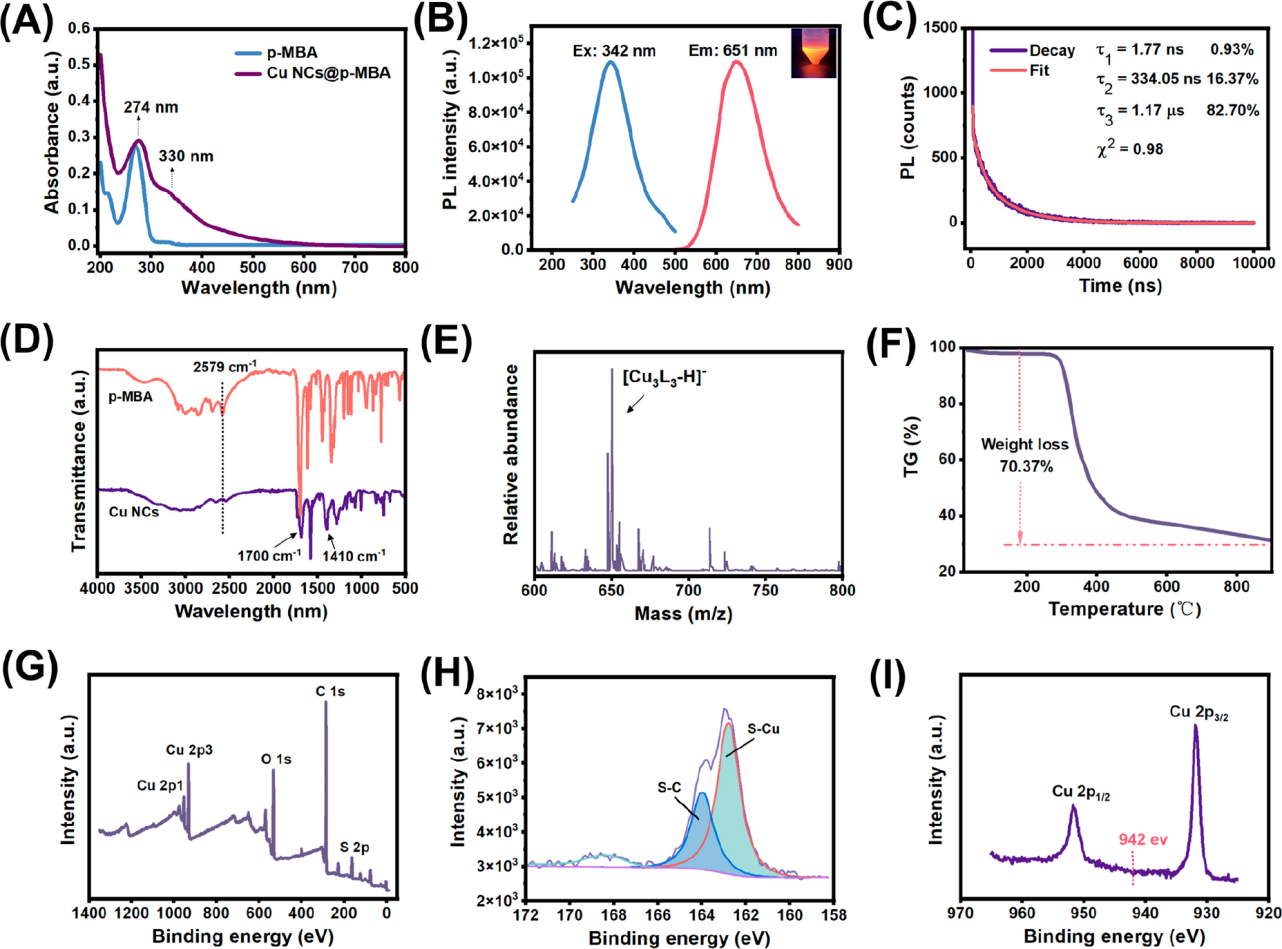
(A) UV–vis absorption spectra of *p*-MBA (blue line) and Cu NCs@*p*-MBA (purple line). (B) The fluorescence excitation (blue line) and emission (red line) spectra of Cu NCs@*p*-MBA (inset: the photograph of Cu NCs@*p*-MBA under UV light). (C) The PL lifetime spectra of Cu NCs@*p*-MBA. (D) FT-IR spectra of *p*-MBA (red line) and Cu NCs@*p*-MBA (purple line). (E) ESI-MS spectrum of Cu NCs@*p*-MBA. (F) TGA profile of Cu NCs@*p*-MBA. (G) The full XPS spectrum of Cu NCs@*p*-MBA. (H) S 2p XPS spectrum of Cu NCs@*p*-MBA. (I) Cu 2p XPS spectrum of Cu NCs@*p*-MBA.

The self-assembled Cu NCs@*p*-MBA synthesized by a simple one-pot method emitted bright orange fluorescence under 365 nm UV light and appeared light yellow under visible light.^26^ In order to obtain Cu NCs@*p*-MBA with the best luminescent properties, the molar ratio of Cu^2+^/*p*-MBA, reaction time, and temperature were optimized to 1:1, 30 min, and 70 °C, respectively, for the following experiments. SEM and AFM images showed that the self-assembled Cu NCs@*p*-MBA were in the form of nanosheets with a width of about 40–90 nm, a length of about 400–800 nm, and a thickness of about 10 nm (Figures S1 and S2). Results from the UV–vis spectroscopy showed two distinct absorption peaks at 274 and 330 nm (Figure 1A). The absorption peaks at 274 nm were assigned to the absorbance of the ligands while the peak at 330 nm was ascribed to the characteristic absorbance of Cu NCs@*p*-MBA arising from the electron transition between the ligands and metal core. Large-sized copper nanoparticles (Cu NPs) have a distinct surface plasmon resonance (SPR) absorption peak in the 400–700 nm region. The absence of this peak provided supporting evidence for the synthesis of Cu NCs@*p*-MBA.^27^

The excitation (Ex) and emission (Em) bands of the fluorescence spectra of Cu NCs@*p*-MBA are centered at 342 and 651 nm, respectively (Figure 1B). A large Stokes shift exceeding 200 nm was evident in the excitation and emission spectra, suggesting that the dominant source of the excited state responsible for emission was primarily of triplet origin.^28^ To elucidate the photoluminescence (PL) emission mechanism of Cu NCs@*p*-MBA, the PL lifetime and quantum yield (QY) were tested. The self-assembled Cu NCs@*p*-MBA (dispersed in ethanol) exhibited an absolute quantum yield (QY) of 11%, which was relatively high compared to other metal nanoclusters.^29−31^ Time-resolved fluorescence spectra showed that the fluorescence lifetime of Cu NCs@*p*-MBA was 1.77 ns (0.93%), 334.05 ns (16.37%), and 1.17 μs (82.70%), with the average lifetime calculated to be 1.019 μs (Figure 1C). The prolonged excited-state lifetime further indicated that the emission from the self-assembled Cu NCs@*p*-MBA may be associated with radiative relaxation of a triplet excited state.^32^ The enhanced emission intensity of the self-assembled Cu NCs@*p*-MBA can be attributed to metal-to-ligand charge transfer (MLCT) or metal-to-metal charge transfer (MMCT) from S atoms in the thiolate ligands to Cu atoms. During the optimization of synthesis conditions, the copper-to-ligand ratio was a critical parameter. Comparative experiments were conducted using different Cu^2+^/*p*-MBA molar ratios (1:1, 1:2, 2:3, and 1:3) to synthesize Cu NCs@*p*-MBA, and their photoluminescence (PL) spectra and fluorescence lifetimes were recorded and are shown in Figure S3A and S3B. The highest emission intensity was obtained with the Cu^2+^/*p*-MBA ratio of 1:1. The fluorescence lifetimes varied slightly in the control experiments and remained in the range of 0.9 to 1 μs, consistent with the emission from triplet states.

The spectra of Fourier transform infrared spectroscopy (FT-IR) further revealed the surface groups of Cu NCs@*p*-MBA. As shown in Figure 1D, the S–H stretching vibration mode (2579 cm^–1^) of the ligand (*p*-MBA) disappeared in the FT-IR spectrum of the synthesized Cu NCs@*p*-MBA, indicating the binding of ligands to Cu atoms via Cu–S covalent bonds. The strong bands at 1700 and 1410 cm^–1^ were related to C=O stretch mode and O–H single bond stretching vibration of the protonated carbonyl (COOH), which provided the sites for supramolecular interactions (hydrogen bonds) in the self-assembled Cu NCs@*p*-MBA.^14^

To determine the exact number of Cu atoms and ligands in the cluster core,^33^ Cu NCs@*p*-MBA were investigated using the ESI-MS measurement in negative-ion mode (Figure 1E). The dominant *m*/*z* peak at 650.49 in the mass spectrum was ascribed to the molecular formula [Cu_3_(L)_3_ – H]^−^ with a theoretical *m*/*z* of 651.00 and deviation of 0.51, where L = C_7_H_5_O_2_S. Further confirmation came from a thermogravimetric analysis (TGA),^34,35^ which exhibited the ligand-to-Cu weight ratio in Cu NCs@*p*-MBA (Figure 1F). The thermogravimetric analysis (TGA) curve indicated a weight loss of 70.37% for *p*-MBA while the calculated value was 70.51%, which aligned with the composition of Cu_3_(L)_3_.

The chemical states of Cu and *p*-MBA in Cu NCs@*p*-MBA were investigated by X-ray photoelectron spectroscopy (XPS). The spectrum showed major peaks for all expected elements of C (1s), O (1s), S (2p), and Cu (2p) (Figure 1G). The S 2p spectrum exhibited two characteristic peaks at 164 and 162.5 eV for the binding energy of C–S and Cu–S bonding (Figure 1H). This was in accordance with the typical value of chemisorption for species S, further confirming that *p*-MBA acted both as a reducing agent and as a capping agent.^28^ The binding energy of Cu 2p appeared at 932 and 951.6 eV, which was assigned to Cu 2p_3/2_ and Cu 2p_1/2_, respectively (Figure 1I). The absence of characteristic satellite peaks at 942 eV indicated that the cluster was mainly composed of Cu(I) and Cu(0) and that no Cu(II) existed. This was consistent with the mild reducing environment corresponding to the weak reducing agent of *p*-MBA and small clusters with sizes of 3–6 atoms.^36^ SEM-EDX analysis proved that the molar ratio of Cu/S was 2.71:2.59, matching the inferred Cu/S ratio of Cu_3_(L)_3_ (Figure S4).

In previous studies, ultrasmall Cu NCs suffered from serious stability issues, limiting their applications in sensing.^37^ In this study, the synthesized self-assembled Cu NCs@*p*-MBA aggregates showed negligible alterations after being stored at 4 °C for 3 months. As shown in Figure S5A, the PL intensity of Cu NCs decreased only slightly, and SEM images showed no change in the morphology of the self-assembled Cu NCs@*p*-MBA before and after storage (Figure S5C). The stability of the sensing labels loaded with Cu NCs@*p*-MBA was further investigated. After being stored in hermetic bags for 1 month at 4 °C, the labels still glowed brightly with no obvious changes (Figure S5B). The long-term stability of Cu NCs@*p*-MBA is attributed to the formation of the aggregate structure of dense nanosheets and the entanglement of ligands of Cu NCs@*p*-MBA within the nanosheets, which protects the luminescent core.^15^

**Figure 2:**
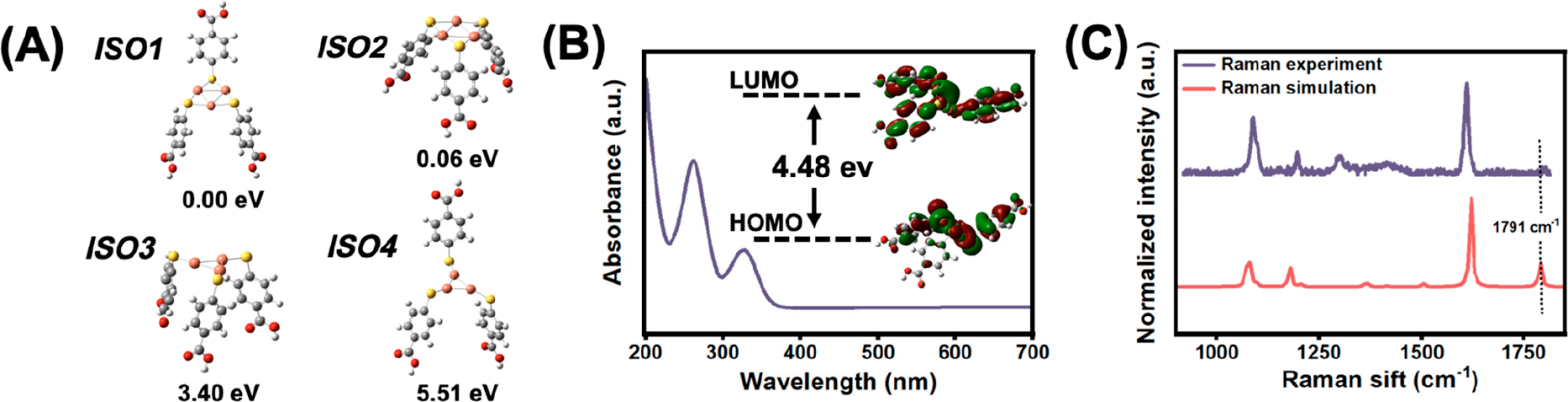
(A) Four isomers of Cu NCs@*p*-MBA and their relative energies. (B) Theoretical UV–vis absorbance spectrum of Iso1 of Cu NCs@*p*-MBA. (C) Simulated Raman spectrum of Iso1 of Cu NCs@*p*-MBA (red line) and experimental Raman spectrum of the self-assembled Cu NCs@*p*-MBA (purple line).

First-principles calculations were carried out to investigate the geometry of Cu NCs@*p*-MBA. The relative energies and isomers of DFT-optimized Cu NCs@*p*-MBA are shown in Figure 2A. According to the combination patterns of Cu atoms and S atoms, the isomers could be further divided into the following two categories: the S atom of the ligand *p*-MBA combined with the adjacent two Cu atoms of the Cu_3_ core (Iso1, Iso2), and the S atom of the ligand *p*-MBA connected to a single Cu atom in the Cu_3_ core (Iso3, Iso4). The simulated spectra of the lowest-energy first group of isomers were further explored. The UV–vis absorption spectra, simulated using time-dependent density functional theory (TD-DFT),^38^ revealed that the theoretically calculated excited states (300–330 nm) of Iso1 closely corresponded to the range of experimentally measured values (Figure 2B). This absorption peak originated from the transition from the S–Cu hybrid bond orbital to the Cu–Cu hybrid bond orbital.^39^ The simulated Raman spectrum of a single Cu NCs@*p*-MBA by frequency calculation is shown in Figure 2C, which was basically consistent with the experimentally measured Raman peaks of the self-assembled Cu NCs@*p*-MBA aggregates, except for the missing peak at 1791 cm^–1^. In the aggregated state, the carbonyl stretching peak (1791 cm^–1^) was weakened by the assembly of single Cu NCs@*p*-MBA through intermolecular interaction. This further confirmed the proposed Cu_3_L_3_ formula.

### 3.2 Self-Assembled Structure and AIE Properties of Cu NCs@*p*-MBA

**Figure 3:**
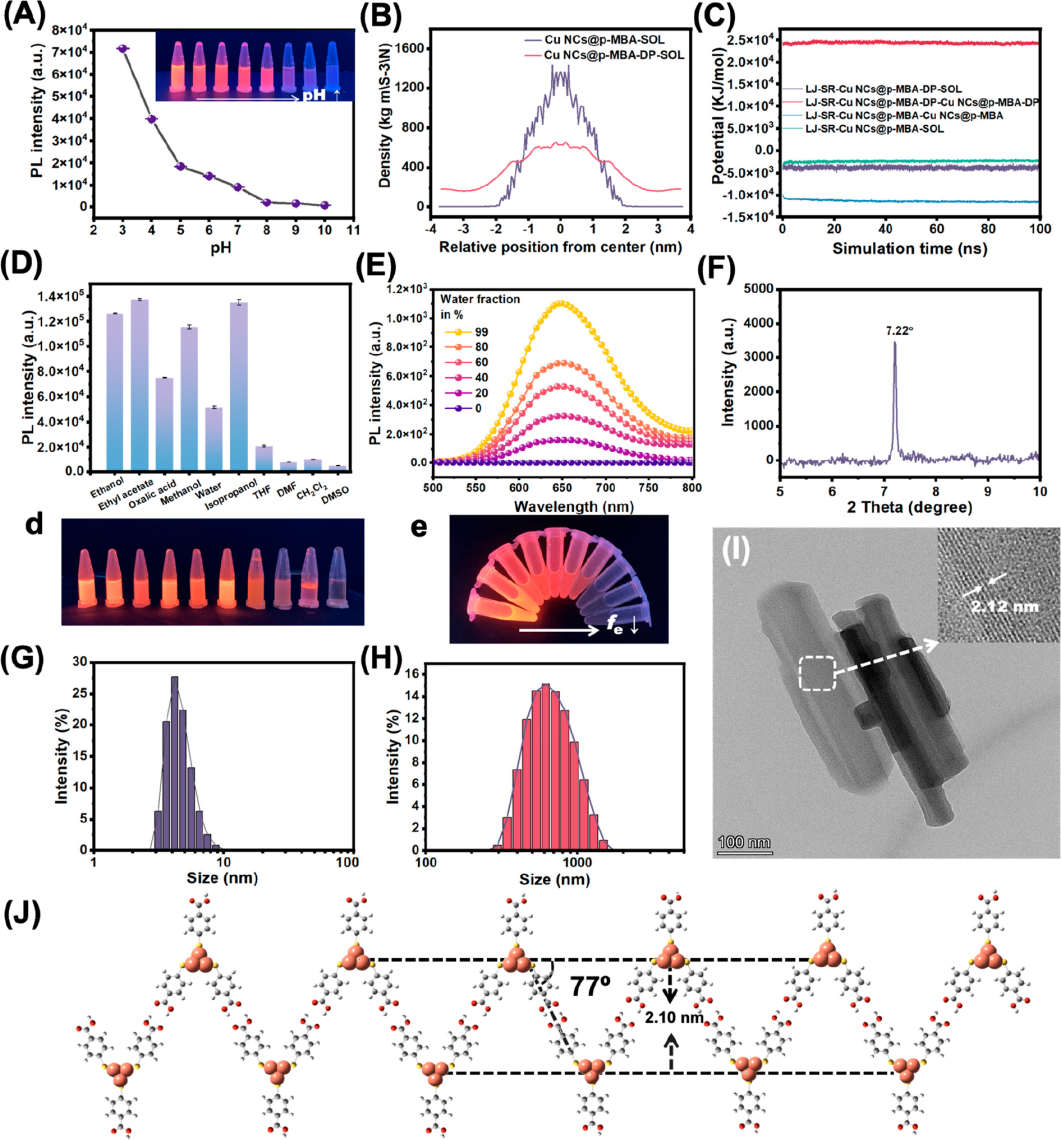
(A) The fluorescence emission spectrum of Cu NCs@*p*-MBA under different pH values (inset: the photograph of Cu NCs@*p*-MBA in pH 3–11 solutions (left to right) under UV light). (B) The density distribution maps of the self-assembled Cu NCs@*p*-MBA and deprotonated Cu NCs@*p*-MBA in a cubic box measuring 7 × 7 × 7 nm in the MD simulation. (C) MD simulation of Lennard–Jones (LJ) potential energies of cluster–cluster and cluster–solvent interactions in acidic and alkaline environments. (D) The PL spectra and (d) fluorescence photograph of Cu NCs@*p*-MBA dispersed in various solvents (i.e., EtOH, EtOAc, oxalic acid, MeOH, water, IPA, THF, DMF, CH_2_Cl_2_, and DMSO). (E) The fluorescence emission spectra of Cu NCs@*p*-MBA in mixed solvents of water and DMSO with increasing *f*_e_ (inset e: the fluorescence photograph of Cu NCs@*p*-MBA in mixed solvents with different *f*_e_ under UV light). (F) SXRD pattern of the self-assembled Cu NCs@*p*-MBA. (G) The DLS of Cu NCs@*p*-MBA in DMSO (*f*_e_ = 0%). (H) The DLS of Cu NCs@*p*-MBA in water (*f*_e_ = 99%). (I) HRTEM image of the self-assembled Cu NCs@*p*-MBA displaying the lattice in the assembled conformation. (J) The arrangement of the self-assembled Cu NCs@*p*-MBA on the 2D plane.

The impact of supramolecular self-assembly on the photoluminescence (PL) of emitters is widely recognized, as it can significantly modify the internal structure of aggregates.^40^ The emission of individual Cu NCs was very weak, and the self-assembled Cu NCs forming a highly ordered internal structure were accompanied by fluorescence enhancement.^13^ First, the AIE characteristics of Cu NCs@*p*-MBA were verified by adjusting the pH value of the system. From pH 3 to 11, the solution changed from turbid to transparent, accompanied by a gradual decrease in PL intensity, with the fluorescence varying from bright orange-yellow, orange-red, and light pink to no fluorescence (Figure 3A). It was evident that the luminescent properties and aggregation degree of Cu NCs@*p*-MBA were highly dependent on pH. In high pH solutions, the ligand *p*-MBA exhibited deprotonated and highly charged carboxylic acid (COOH) groups. Cu NCs@*p*-MBA repelled each other and dissolved as isolated species. Upon decreasing the pH value, the protonated Cu NCs@*p*-MBA were transformed from disordered dispersion to ordered aggregation, which further limited the intramolecular vibration and rotation of the ligand *p*-MBA, prevented the nonradiative decay process of the excited state, and achieved strong emission. In addition, the aggregation of Cu NCs@*p*-MBA also promoted Cu(I)···Cu(I) interactions between clusters, and the enhanced coprophilic interaction provided a driving force for aggregation and dramatically improved the radiation relaxation of excited states.^11^

The distribution and packing of Cu NCs@*p*-MBA were visualized through the density distribution of molecular dynamics (MD) simulation, providing further confirmation of their dispersion characteristics under different pH environments. The degree of dispersion of Cu NCs@*p*-MBA in acidic environments was significantly lower than that of deprotonated Cu NCs@*p*-MBA in alkaline environments (Figure 3B). The analysis of the Lennard–Jones (LJ) potential energy in the MD simulations explained the contribution of the nonelectrostatic interactions between molecules in the system to the overall stability of the assembled nanostructures. As shown in Figure 3C, there were repulsive interactions between deprotonated Cu NCs@*p*-MBA. In acidic environments, the LJ potential energy indicated the presence of attractive forces between clusters that promoted aggregation. Besides, the LJ potential energy between Cu NCs@*p*-MBA and solvent molecules was found to be significantly smaller compared to the potential energy between the clusters themselves. This observation provided strong evidence for the formation of nanosheets, as it indicated that the interaction forces between the clusters were much stronger than those between the clusters and the solvent molecules. Consequently, the nanosheets exhibited limited dispersion in the solution, ensuring their structural integrity and stability.

The self-assembly behavior and AIE characteristics of Cu NCs@*p*-MBA could also be manipulated by adjusting the interactions between structural units and solvents. Cu NCs@*p*-MBA were dispersed in various solvents, including water, EtOH, MeOH, DMF, THF, and DMSO, to observe the photoluminescence intensity and solubility further. As shown in Figure 3D, Cu NCs@*p*-MBA completely dissolved and showed weak fluorescence in a protonated solvent (DMSO) but gave off a bright orange color under UV light when protonated solvents such as water, EtOH, and MeOH were added. The AIE properties of Cu NCs@*p*-MBA in the mixed solvents of DMSO and water were further studied by fluorescence spectroscopy and DLS measurements. The degree of aggregation and fluorescence intensity increased with the volume fraction of water in the solvent (*f*_e_ = vol_waterl_/vol_water_ + vol_DMSO_). The solvent mixture (*f*_e_ = 99%) exhibited the desired strong emission (Figure 3E). With the increase of *f*_e_ from 0% to 99%, DLS measurement showed that the Cu NCs@*p*-MBA gradually aggregated from a monodisperse state (3 to 5 nm) into nanosheets (700 nm) (Figure 3G and 3H).

HRTEM images presented the nanosheet structure composed of a single Cu NC@*p*-MBA in an aggregated state. The self-assembled Cu NCs@*p*-MBA exhibited highly ordered layered features with an interlayer distance of 2.12 nm, setting them apart from the facet lattice observed in crystalline Cu (111) (Figure 3I).^15^ This ordered arrangement was further verified by the intense peaks observed in the small-angle X-ray diffraction (SXRD) pattern, as shown in Figure 3F. Specifically, there was a distinct diffraction peak observed at 7.22°, indicating a *d*-spacing of 2.12 nm and revealing the interlayer distance within the assembled nanosheets. The arrangement of nanosheets on the two-dimensional plane was further provided. In the stable structure calculated by the Gaussian 16 program package, the size of Cu NCs@*p*-MBA and the length of the ligand were 1.91 and 1.06 nm, respectively. The configurations of the assembled Iso1 are depicted in Figure 3J, and the distances between the layers were consistent with the findings observed through HRTEM and XRD.

### 3.3 Sensitivity and Selectivity of the Sensing System

**Figure 4:**
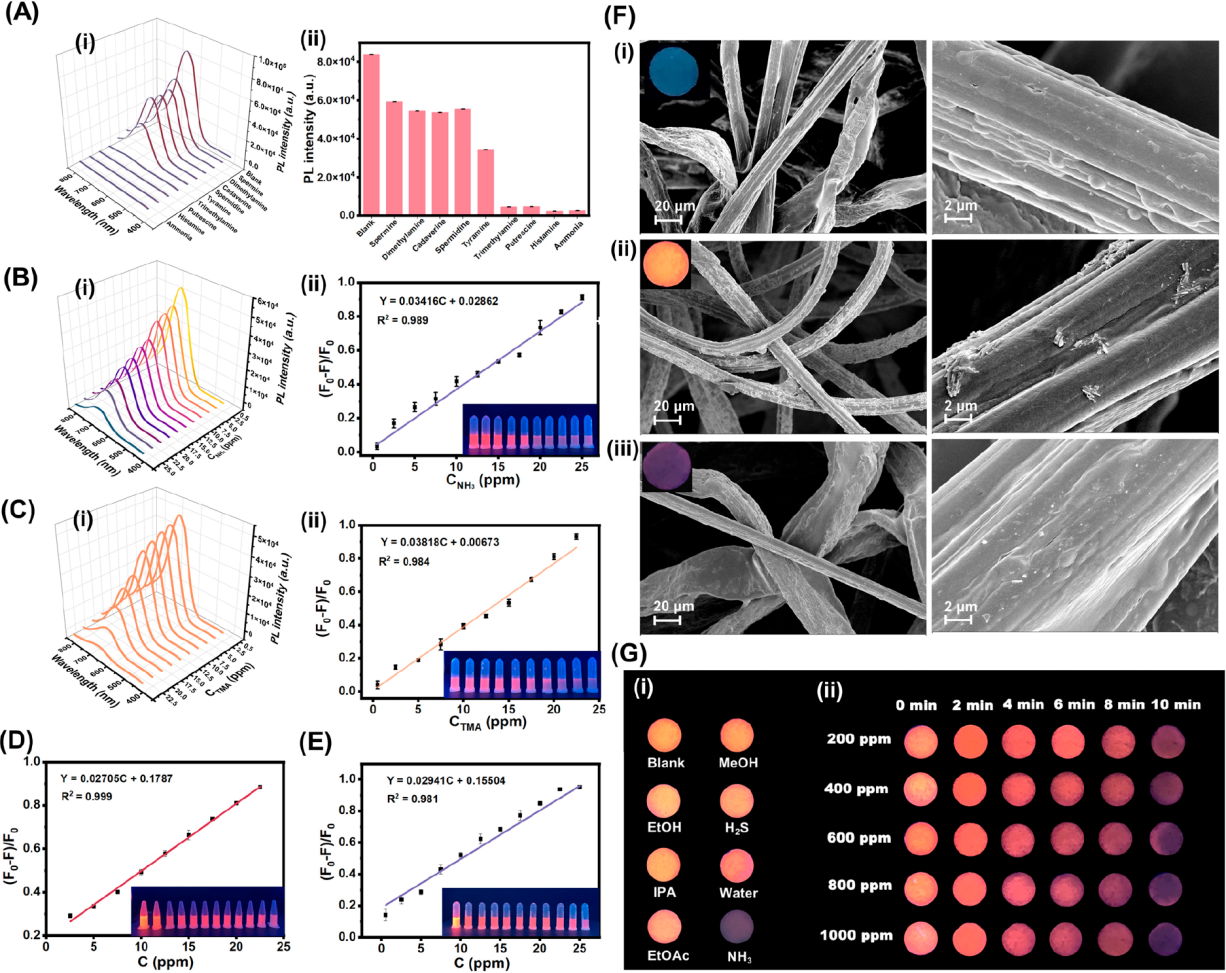
(A) The fluorescence response of the self-assembled Cu NCs@*p*-MBA to different VBNs (50 ppm of spermine, cadaverine, spermidine, tyramine, putrescine, histamine, dimethylamine, trimethylamine, and ammonia). (B) The fluorescence response of the self-assembled Cu NCs@*p*-MBA to different concentrations of ammonia (0.5, 2.5, 5.0, 7.5, 10.0, 12.5, 15.0, 17.5, 20.0, 22.5, and 25.0 ppm), including (i) the fluorescence emission spectra recording the change of the PL intensity, and (ii) the linear relationship of *F*_0_ – *F*/*F*_0_ versus the concentrations of ammonia. (C) The fluorescence response of the self-assembled Cu NCs@*p*-MBA to different concentrations of trimethylamine from 0.5 to 25 ppm, including (i) the fluorescence emission spectra recording the change of the PL intensity; (ii) the calibration curve calculated from *F*_0_ – *F*/*F*_0_ versus the concentration of trimethylamine. (D) The calibration curve calculated from *F*_0_ – *F*/*F*_0_ versus the concentration of putrescine. (E) The calibration curve calculated from *F*_0_ – *F*/*F*_0_ versus the concentration of histamine. (F) SEM images of the fluorescent labels, including (i) the cellulose cotton paper discs, (ii) the sensing labels loaded with the self-assembled Cu NCs@*p*-MBA nanosheets, and (iii) the sensing labels after reacting with gaseous ammonia. (G) The fluorescence photographs of the sensing labels after reacting with different volatile compounds: (i) exposed to 2000 ppm of H_2_S, MeOH, EtOH, IPA, EtOAc, IPA, NH_3_, and H_2_O; (ii) reacting with different concentrations of ammonia vapor (0–1000 ppm) within 10 min.

During the spoilage process, fish release unpleasant odors containing a variety of volatile basic nitrogens (VBNs), including ammonia (NH_3_), dimethylamine (DMA), trimethylamine (TMA), and biogenic amines.^3^ To investigate the sensitivity of Cu NCs@*p*-MBA in detecting spoilage markers, model targets including ammonia, dimethylamine, trimethylamine, and a range of biogenic amines (histamine, cadaverine, putrescine, spermine, spermidine, and tyramine) were selected, with water as the blank control. Fluorescence spectroscopy was utilized to monitor the response of the prepared Cu NCs@*p*-MBA toward various amine species, with particularly high sensitivity observed for ammonia, putrescine, and trimethylamine, which are the primary components of TVB-N^41^ (Figure 4A). This indicated the applicability and accuracy of Cu NCs@*p*-MBA for practical seafood monitoring applications. Subsequently, the fluorescence response of ammonia, trimethylamine, and putrescine at different concentrations was studied. The fluorescence intensity of the self-assembled Cu NCs@*p*-MBA aggregates showed a gradual reduction with the increase of ammonia concentration, in the range of 0.5 to 25 ppm (Figure 4B). The fluorescence intensities *F*_0_ – *F* (*F*_0_ and *F* were the luminous intensity of Cu NCs@*p*-MBA at 651 nm in the absence and presence of the analyte, respectively) versus ammonia concentrations performed a good linear relationship (*R*^2^ = 0.989), with a limit of detection (LOD) value of 0.33 ppm (S/N = 3). For trimethylamine, a linear calibration curve with the concentration of trimethylamine was established (i.e., (*F*_0_ – *F*)/*F*_0_) and the LOD was calculated to be 0.81 ppm (S/N = 3) (Figure 4C). For putrescine detection, as shown in Figure 4D, there was a linear correlation between the fluorescence intensity and putrescine concentration within the range of 2.5 to 25 ppm, yielding the LOD of 1.58 ppm.

In the case of biogenic amines, Cu NCs@*p*-MBA displayed a high sensitivity to heterocyclic histamine but exhibited a weaker quenching effect when exposed to aromatic amines^42^ (Figure 4A). The reduced responsiveness to aromatic amines may be attributed to the electron delocalization of the nitrogen atom into the aromatic ring, resulting in reduced basicity and nucleophilicity of aromatic amines.^43^ Due to the presence of the electron-rich imidazole moiety in histamine, it was hypothesized that a strong interaction between the metal core of Cu NCs@*p*-MBA and the imidazole group led to fluorescence quenching, hence demonstrating high sensitivity.^15^ As depicted in Figure 4E, the calibration plot between the quenching efficiency and different histamine concentrations displayed a good linear relationship with the LOD of 1.01 ppm. Therefore, the results suggested that the self-assembled Cu NCs@*p*-MBA aggregates exhibited high sensitivity to amine species, indicating their potential to detect spoilage markers.

The fluorescence response of fiber cotton discs containing the self-assembled Cu NCs@*p*-MBA to VBNs was further investigated. First, the morphology of the cellulose cotton paper substrate, as well as the fluorescence sensing labels loaded with Cu NCs@*p*-MBA before and after reacting with ammonia, was characterized using scanning electron microscopy (SEM) (Figure 4F). As shown in Figure 4F(i), the cotton paper used as the blank control did not emit any fluorescence, and a loose porous fiber structure was observed. There was no obvious attachment on the rough fiber surface. A large number of nanosheets appeared on the surface of the fiber of labels loaded with Cu NCs@*p*-MBA, confirming the successful fabrication of fluorescent labels (Figure 4F(ii)). The cotton paper reacted with ammonia showed no fluorescence, and no large-area aggregates were observed on the surface of a single fiber (Figure 4F(iii)). Next, the labels were exposed to varying concentrations of ammonia vapor (0–1000 ppm), and the fluorescence changes were recorded every minute. As shown in Figure 4G(ii), with the increase of ammonia concentration and time, a gradient change of fluorescence color from orange-yellow, orange-red, and pink to no fluorescence was observed. These results indicated that the facilely prepared fluorescent labels exhibited unique fluorescent responses to amines and had great potential for noncontact seafood spoilage monitoring.

Subsequently, the efficacy of the sensing system was evaluated by investigating its response toward various volatile compounds during seafood spoilage, including H_2_S, MeOH, EtOH, IPA, EtOAc, IPA, and H_2_O. As depicted in Figure 4G(i), the labeling agents did not exhibit any fluorescence response upon exposure to the aforementioned volatile compounds, which signified the high selectivity of Cu NCs@*p*-MBA in detecting VBNs.

### 3.4 Detection Mechanism of the Sensing System

**Figure 5:**
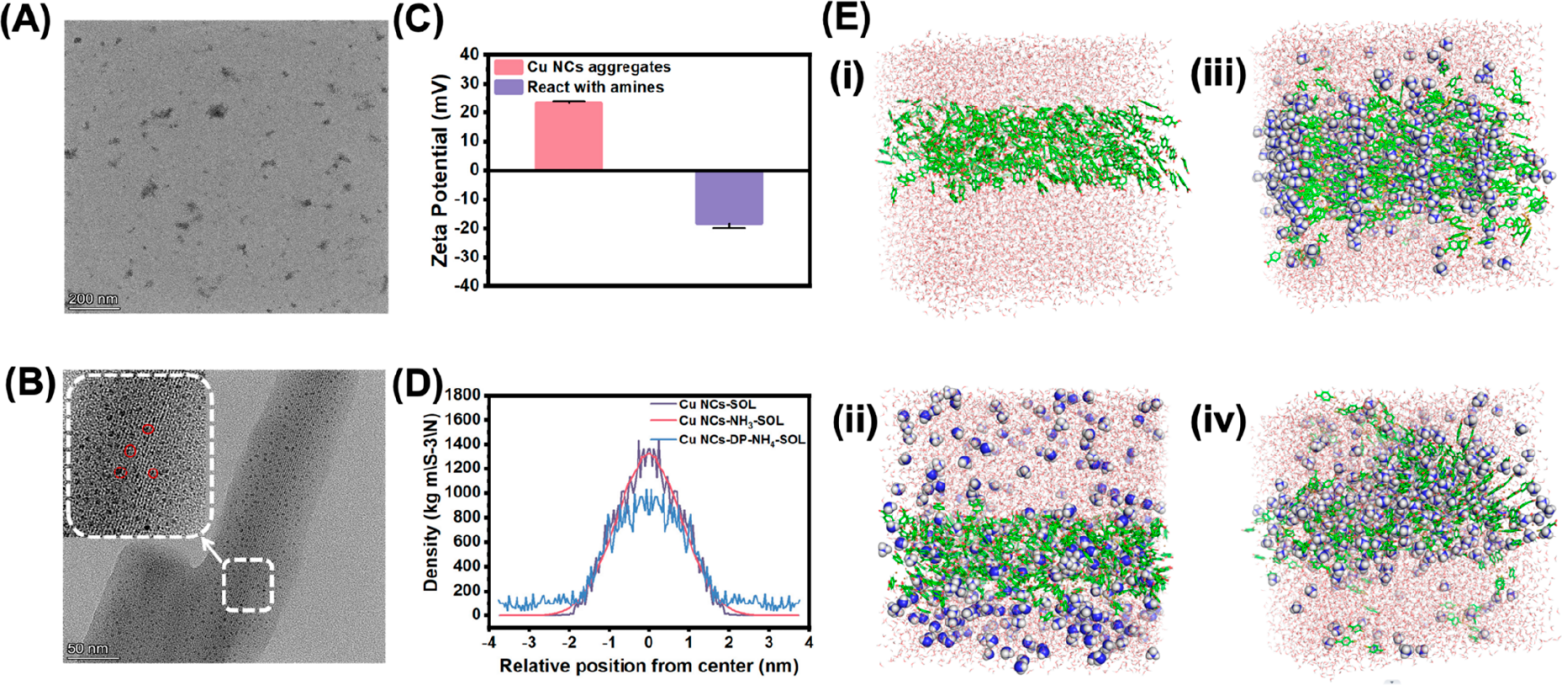
(A) TEM image of the self-assembled Cu NCs@*p*-MBA after adding 100 ppm ammonia. (B) HRTEM image of the nanosheets of Cu NCs@*p*-MBA in the presence of 10 ppm ammonia. (C) Zeta potential of the self-assembled Cu NCs@*p*-MBA before and after the reaction with ammonia. (D) The density analysis plots of the self-assembled Cu NCs@*p*-MBA, as well as CuNCs@*p*-MBA, in contact with ammonia and subsequently reacted with ammonia in the MD simulation. (E) The renderings of the distribution of (i) the self-assembled Cu NCs@*p*-MBA, (ii) the self-assembled Cu NCs@*p*-MBA upon contact with ammonia molecules at 0 ns, and their subsequent reaction with ammonia at 50 (iii) and 100 ns (iv).

In order to gain a deeper understanding of the high sensitivity of the self-assembled Cu NCs@*p*-MBA in detecting amines, the sensing mechanism was explored. TEM images revealed the morphology of the self-assembled nanosheets of Cu NCs@*p*-MBA after being sufficiently exposed to ammonia. The originally compact and ordered nanosheet structure was significantly disrupted, and a considerable number of spherical particle fragments were observed (Figure 5A). Upon introducing low concentrations of ammonia, a small number of sheet-like aggregates could still be observed, but the layered crystalloid structure of the nanosheets experienced the emergence of numerous 3–4 nm nanospherical substances, leading to the destruction of the lattice between the crystal planes (Figure 5B). On the basis of these observations, it was speculated that the noncovalent interaction forces (hydrogen bonds) were weakened. Zeta (ζ) potential measurements indicated that the ζ potential value of Cu NCs@*p*-MBA after the reaction with ammonia changed from positive (23.3 mV) to negative (−31.2 mV), which may be due to the deprotonation of the COOH group on the surface of the ligand in a weakly basic environment (Figure 5C). This was consistent with the behavior of Cu NCs@*p*-MBA in high pH, in which the supramolecular driving force weakened, ultimately leading to the breakdown of the ordered structure.

**Figure 6:**
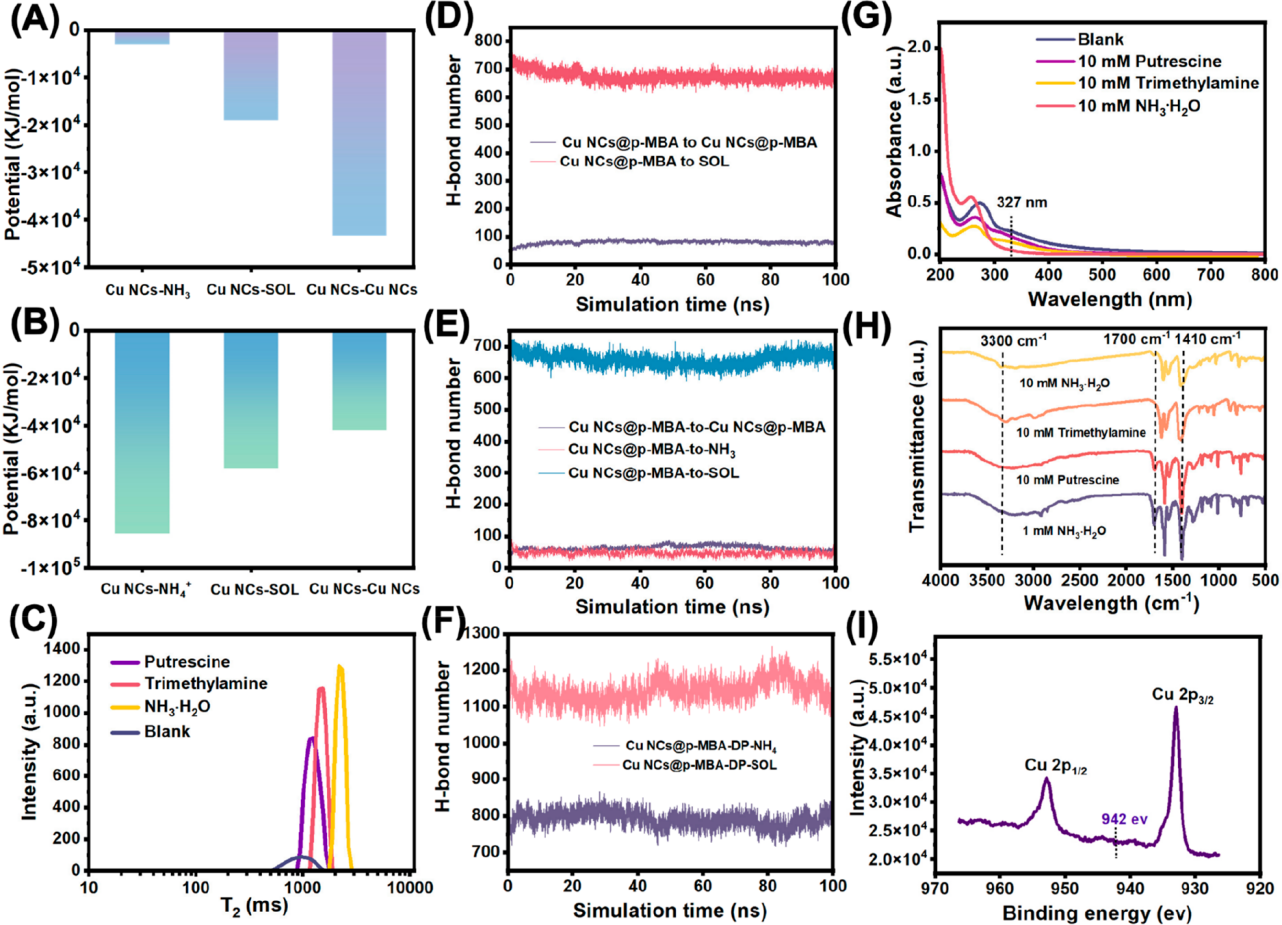
(A) MD simulation of Coulomb forces of cluster–NH_3_ interactions, cluster–solvent interactions, and cluster–cluster interactions of CuNCs@*p*-MBA in contact with ammonia. (B) MD simulation of Coulomb forces of the interactions between cluster–NH_4_^+^, cluster–solvent, and cluster–cluster in the sensing system of CuNCs@*p*-MBA after reacting with ammonia. (C) The *T*_2_ relaxation times of LF-NMR for the self-assembled Cu NCs@*p*-MBA with the addition of putrescine, trimethylamine, and ammonia. (D) The dynamic evolution of hydrogen bonds in the self-assembled Cu NCs@*p*-MBA nanosheets during the 0–100 ns simulation. (E) Changes in the number of hydrogen bonds between cluster–cluster, cluster–NH_3_, and cluster–solvent after the introduction of ammonia under the dynamic simulation of 0–100 ns. (F) The dynamic change of hydrogen bonds between deprotonated Cu NCs@*p*-MBA and ammonium from 0 to 100 ns following the reaction of Cu NCs@*p*-MBA with ammonia. (G) UV–vis absorption spectra of the self-assembled Cu NCs@*p*-MBA after adding putrescine, trimethylamine, and ammonia. (H) FT-IR spectra of the self-assembled Cu NCs@*p*-MBA in the presence of different concentrations of putrescine, trimethylamine, and ammonia. (I) Cu 2p XPS spectrum of the self-assembled Cu NCs@*p*-MBA after adding ammonia.

Molecular dynamics (MD) simulations were conducted to investigate the density distribution and Coulomb forces during the reaction to further support the findings from TEM images and zeta potential results. Initially, highly luminescent Cu NCs@*p*-MBA in the form of compact nanosheets were constructed within a cubic box measuring 7 × 7 × 7 nm in the MD simulations. Figure 5E illustrates the structural changes of the nanosheets upon contact with ammonia molecules at 0 ns and their subsequent reaction with ammonia at 50 and 100 ns, respectively. With the progression of the reaction, the dense nanosheets gradually disassembled. The density analysis plots revealed that the dispersion of fully reacted nanosheets with ammonia exceeded that of nanosheets in contact with ammonia but was lower than that of deprotonated Cu NCs@*p*-MBA in a highly alkaline environment (Figure 5D). The pH of the sensing system of Cu NCs@*p*-MBA reacted with 50 ppm ammonia increased from 3 to 5, and the degree of fluorescence quenching aligned with that observed in a highly alkaline environment (pH = 11). In combination with the dispersion degree in the solution, it was demonstrated that the sensitivity of Cu NCs@*p*-MBA toward ammonia molecules surpassed their sensitivity to pH. The Coulomb forces calculated by the Gromacs 2019.1 software package represented the electrostatic interactions between charged particles within the system. As depicted in Figure 6A and Figure 6B, in the early stages of sensing, the most dominant electrostatic interactions were observed between Cu NCs@*p*-MBA themselves, exceeding the interactions with the solvent and ammonia molecules. As the reaction progressed, the electrostatic interaction between Cu NCs@*p*-MBA and ammonium ions became the prevailing force, surpassing the interactions between clusters. The change in the electrostatic interactions was consistent with the density distribution during the sensing process.

Low-field nuclear magnetic resonance (LF-NMR) was used to study the *T*_2_ relaxation time of the self-assembled Cu NCs@*p*-MBA reacting with different amines, including putrescine, trimethylamine, and ammonia (Figure 6C). The relaxation speed could reflect the motion environment of the hydrogen nucleus from a microscopic point of view.^44^ In the range of 500–1000 ms, the relaxation time and peak strength of Cu NCs@*p*-MBA with amines increased, possibly due to the release of highly bound hydrogen nuclei in the compact supramolecular structure. From a nuclear magnetic mechanism perspective, the binding effect between Cu NCs@*p*-MBA in the aggregate was comparatively strong, resulting in weaker Brownian motion in comparison to that observed in Cu NCs@*p*-MBA reacting with ammonia. Consequently, the transverse relaxation time was relatively shorter in the former case. Besides, the relaxation time increase of the self-assembled Cu NCs@*p*-MBA with ammonia was greater than that with trimethylamine and putrescine. This was consistent with the sequence of PL intensity reduction, suggesting that the self-assembled Cu NCs@*p*-MBA could be more sensitive to ammonia with a small molecular weight.

Using the Gromacs 2019.1 software package, the dynamics of hydrogen bond formation within the system were investigated. Initially, the number of hydrogen bonds between clusters in the aggregates increased over time and reached a stable range of 80–90 bonds (Figure 6D). Upon the introduction of ammonia molecules, the total number of hydrogen bonds between clusters decreased to 50–60, while the number of hydrogen bonds between clusters and ammonia reached a steady state of approximately 50 within the 0–100 ns simulation time (Figure 6E). This suggested that ammonia molecules partially disrupted the existing tight hydrogen bond associations within the aggregates and formed new hydrogen bonds with a subset of the clusters. Subsequently, as the ammonia molecules further reacted, the number of hydrogen bonds between ammonium ions and clusters increased significantly (Figure 6F). In the visualization image, the formation of scattered aggregate was observed, consistent with the experimental observation of solution clarity and the disappearance of fluorescence.

The UV–visible absorption spectra indicated that the distinct peaks of Cu NCs@*p*-MBA at 327 nm gradually vanished when exposed to VBNs, as demonstrated in Figure 6G. The peak at 327 nm was typically associated with the electron transition between the ligands and the metal core. This result suggested that the fluorescence quenching of Cu NCs@*p*-MBA may occur through an electron transfer mechanism as a result of the electrostatic interaction between VBNs and the ligands. The changes in the surface groups of Cu NCs@*p*-MBA during the reaction with various VBNs were measured via FT-IR spectra (Figure 6H). With increasing concentration of amine species, the two groups of characteristic peaks related to hydrogen bond formation gradually weakened. These included the strong C=O stretching peak (1700 cm^–1^) and the distinctive absorption peak corresponding to the stretching of the OH single bond (1410 cm^–1^). Simultaneously, the intensity of the N–H stretching vibration absorption peak (2900–3300 cm^–1^) increased. Additionally, no characteristic satellite peaks of Cu(II) were observed in the Cu 2p XPS spectrum (Figure 6I), indicating that the addition of NH_3_ did not alter the oxidation state of Cu atoms.

Obviously, the self-assembled supramolecular structure of Cu NCs@*p*-MBA changed after adding amines. In aggregates of Cu NCs@*p*-MBA, the benzoic acid moiety of the ligand that provided the driving force for supramolecular assembly also had a strong affinity for amines. The hydrogen bonds between the nanoclusters in the aggregates of adsorbed NH_3_ were gradually replaced by the hydrogen bonds between the clusters and the NH_3_. The repulsive force between the Cu NCs@*p*-MBA increased and gradually dispersed. The intramolecular vibrational and rotational motion of the ligand within the dispersed aggregates was enhanced, thereby causing a decrease in PL intensity. Moreover, the interaction between the COOH groups of the self-assembled Cu NCs@*p*-MBA and the N–H groups of the amines exerted a further impact on the electron transfer mechanism. It was postulated that this interaction hindered the LMCT or LMMCT from the S atom in the ligand to the Cu atom.

### 3.5 Real-Time Monitoring of Seafood Spoilage

To evaluate the effectiveness of the prepared fluorescent labels for real-time monitoring of seafood freshness, spoilage experiments were conducted on sea bass fillets at both room temperature (25 °C) and refrigeration temperature (4 °C). The sensing labels were photographed under 365 nm UV light throughout storage to record the fluorescence color changes. The TVB-N value, a standard parameter for evaluating spoilage, was tested to assess the accuracy of the labels.

**Figure 7:**
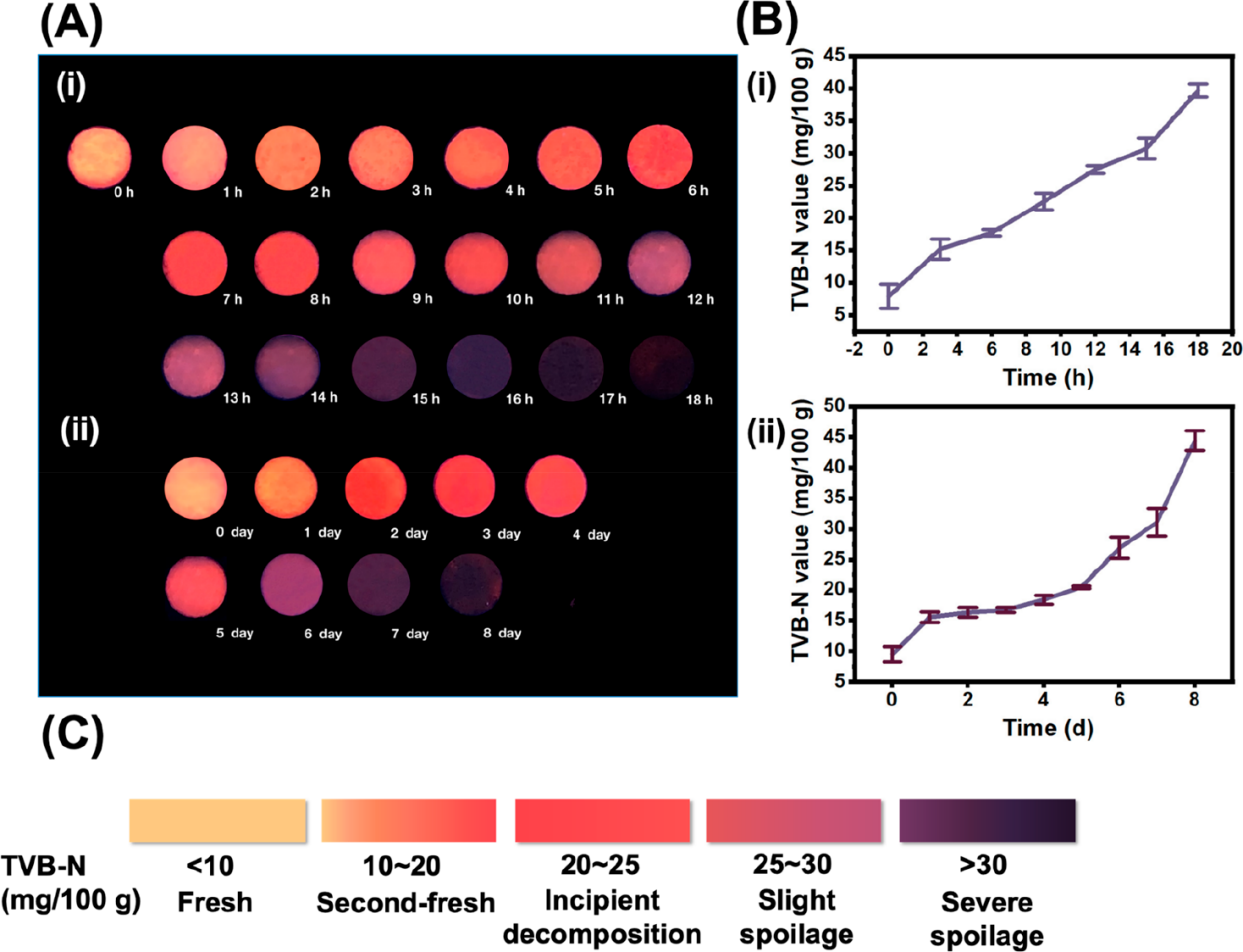
(A) The fluorescent color change of the sensing labels loaded with Cu NCs@*p*-MBA during the monitoring of sea bass freshness at (i) 25 and (ii) 4 °C. (B) The change of TVB-N values in sea bass, stored at (i) 25 and (ii) 4 °C. (C) The created color card reflecting the freshness of the seafood.

The initial TVB-N value of fresh sea bass was 6.78 mg/100 g. Following storage at room temperature for 3 and 6 h, the TVB-N value significantly increased to 15.16 and 17.66 mg/100 g, respectively (Figure 7B(i)). Concurrently, the color of the fluorescent label transformed from bright orange-yellow to orange-red and light red, indicating that the fish had undergone slight decomposition but was still safe for consumption and classified as being in a subfresh state (Figure 7A(i)). After 9 and 12 h storage, the TVB-N levels escalated to 22.59 and 27.51 mg/100 g, respectively, and the fluorescent labels demonstrated colors ranging from rosy red to pink-purple, reflecting different freshness statuses of beginning to rot and slight spoilage. With prolonged storage time, the TVB-N value exceeded 30 mg/100 g, and the fluorescence color gradually evolved from purple to dark purple until the camera could not capture the fluorescence emission, indicating the severe deterioration of the fish.

Under refrigerated conditions, the color change of fluorescent labels could effectively indicate the degree of spoilage of fish. In the initial stage of storage (0–4 d), the TVB-N value increased from 6.54 to 18.41 mg/100 g, while the fluorescent label gradually transformed from orange-yellow to light red, signifying the subfresh state of fish. During the later stage of storage (5–8 d), the fluorescent labels changed from pink-purple, dark purple, and eventually to no fluorescence, corresponding to TVB-N values of 25.61, 31.12, and 35.11 mg/100 g, respectively (Figure 7A(ii) and 7B(ii)). These transformations reflected the progression from beginning to rot, slight spoilage, to severe spoilage.

The above findings confirmed the correlation between the color change of the fluorescent label and the standard TVB-N method, thereby substantiating the applicability of the developed labels for nondestructive and real-time monitoring of seafood freshness. As shown in Figure 7C, the color card was further established, which categorized the fluorescent colors associated with freshness into five levels, namely, orange-yellow (fresh), orange-red (second-fresh), light pink (incipient decomposition), pink-purple (slight spoilage), and deep purple to black (severe spoilage). The development of a standardized color card furnished a more user-friendly modality of visual monitoring for on-site real-time evaluation.

**Figure 8:**
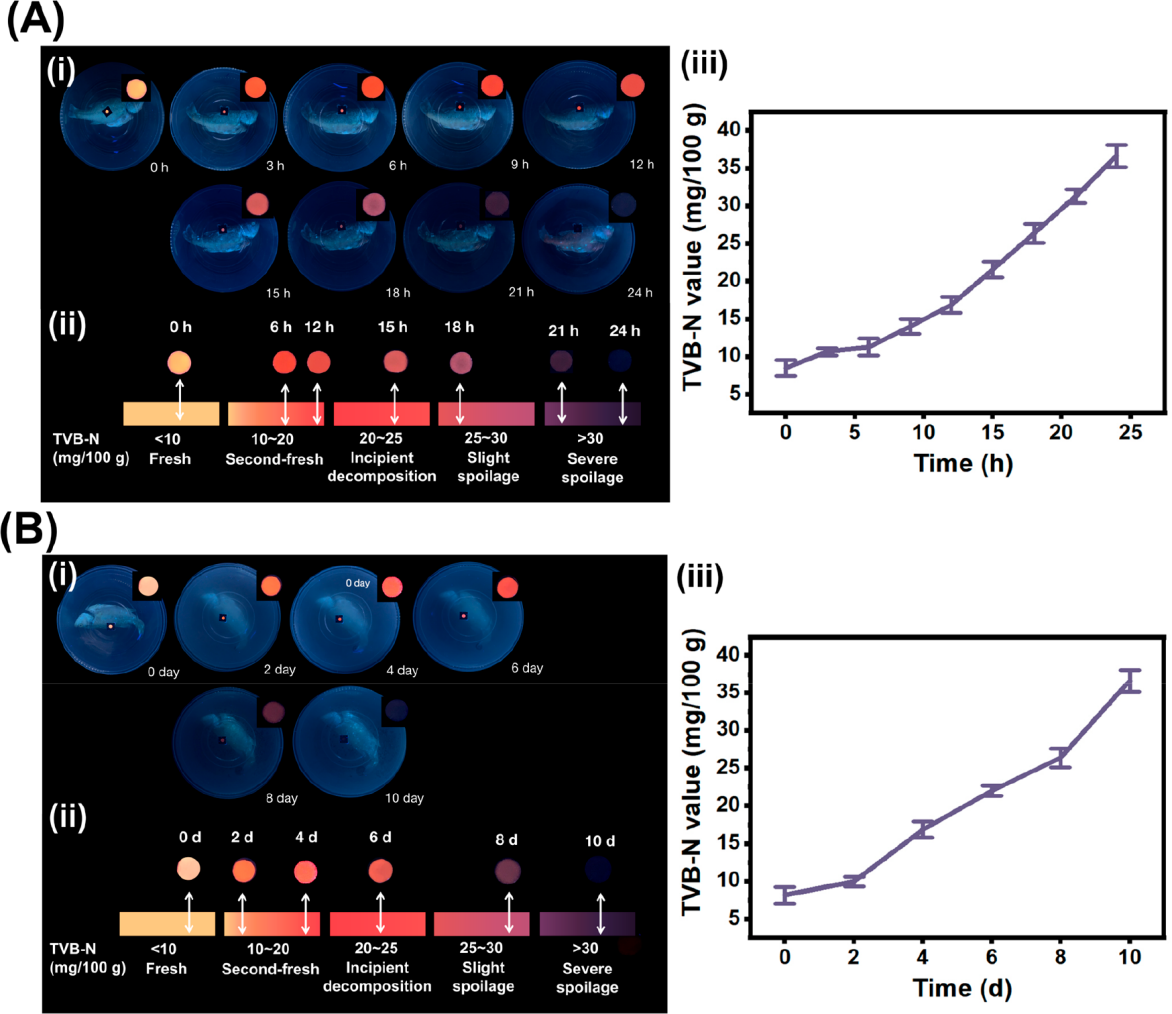
(A) (i) The fluorescent color change of the sensing labels loaded with Cu NCs@*p*-MBA during the monitoring of the whole sea bass freshness at 25 °C. (ii) Correspondence of the created color card and fluorescent label colors at different periods. (iii) The change of TVB-N values in the whole sea bass stored at 25 °C. (B) (i) The fluorescent color change of the sensing labels loaded with Cu NCs@*p*-MBA during the monitoring of the whole sea bass freshness at 4 °C. (ii) Correspondence of the created color card and fluorescent label colors at different periods. (iii) The change of TVB-N values in the whole sea bass stored at 4 °C.

Furthermore, the prepared fluorescent labels were employed to monitor the freshness of whole sea bass samples at 4 and 25 °C. At 25 °C, the complete spoilage of the whole fish was extended to 24 h, while at 4 °C, it took 10 days for complete spoilage to occur. As depicted in Figure 8A and 8B, the use of the established color card to correlate the color responses of the labels during freshness monitoring with the measured TVB-N values yielded consistent results, indicating the good accuracy and versatility of the sensing labels.

The developed sensing method was also compared to several previously reported fluorescent sensors, as shown in Table 1. This study demonstrates the detection ranges and limits that are comparable to or even better than those reported in most previous studies. As previously mentioned, many studies focused on the sensitivity to volatile basic nitrogen (VBNs) in the headspace for freshness detection. However, some sensing probes with aggregation-caused quenching (ACQ) properties were only suitable for sensing in solution systems, which limited their potential for on-site and real-time freshness monitoring.^45,46^ The sensors based on aggregation-induced emission (AIE) were more suitable for constructing solid-state sensors, such as self-assembled films and paper-based analytical devices (PAD), among others. Nevertheless, most organic AIE molecules exhibited specific responses to individual VBNs or selective responses to a subset of VBNs, thereby restricting freshness monitoring to qualitative or quantitative analysis of individual VBNs.^5,15,16^ In this study, the sensing labels, built upon the self-assembled CuNCs@*p*-MBA nanosheets, displayed high sensitivity to the major volatile gases within TVB-N. Compared to AIE-type CuNCs-GSH, the self-assembled CuNCs@*p*-MBA exhibited better sensitivity to low-molecular-weight amines.^27^ Therefore, the high sensitivity and easily distinguishable fluorescence color changes of the labels made them highly suitable for semiquantitative, visual, and real-time monitoring of seafood spoilage.

**Table 1:** Sensing Performance of the Self-Assembled Cu NCs@*p*-MBA Compared with Different Fluorescence Methods Reported in the Literature

material	type	detection mode	solvent/support	monitoring object	detection range	limit of detection	sample	freshnessmonitoring mode	reference
Bp(Im)_2_MA	AIE	gas-phase detection	cotton fiber absorbent pad	putrescine	NA	4.3 ppm	raw fish	qualitative evaluation	(47)
		liquid-phase detection	chloroform (CH_3_Cl)	spermine	0–10 μM	180 nM			
HMBA-4	SAIE	gas-phase detection	self-assembled film	ammonia	0–652 Pa	186 Pa	fresh lean pork	qualitative evaluation	(5)
m-DB	SAIE	gas-phase detection	self-assembled film	ammonia	0.2–3.4 Pa	2.02 Pa	pork	qualitative evaluation	(16)
TPEBA and CPTH	SAIE	gas-phase detection	self-assembled film	trimethylamine	1.7–26.4 ppm	0.89 ppm	butterfish	qualitative evaluation	(6)
TPM @ CB[8]	SAIE	gas-phase detection	glass plate	ammonia	20–500 ppm	0.80 ppm	pork	qualitative evaluation	(48)
HBTAc	AIE	gas-phase detection	filter paper	ammonia	0–300 ppm	12.7 ppm	pomfret	qualitative evaluation	(49)
N,S-CDS	ACQ	liquid-phase detection	ultrapure water	ammonia	2–80 mmol/L	0.005 mmol/L	bighead carps	qualitative evaluation	(46)
CDS	ACQ	liquid-phase detection	ultrapure water	ammonia	0.5–50 mM	NA	tap water and river water	NA	(45)
DPA-CuNPs	SAIE	liquid-phase detection	ultrapure water	histamine	0.05–5 μM	30 nM	fish, pork, and red wine	NA	(50)
TFTP-Cu NCs	SAIE	gas-phase detection	cotton fiber	histamine	0.1–10 μM	60 nM	fish (carp), shrimp, and red wine	quantitative evaluation (histamine)	(15)
Cu NCs-GSH	AIE	gas-phase detection	cotton fiber	TVB-N	10–50 ppm	NH_3_: 1.41 ppm	salmon	semiquantitative evaluation	(27)
Cu NCs@*p*-MBA	SAIE	gas-phase detection	cotton fiber	TVB-N	2.5–25 ppm	NH_3_: 0.33 ppm;TMA: 0.81 ppm	sea bass	semiquantitative evaluation	this work

## 4 Conclusions

In this study, mercaptobenzoic acid-terminated highly luminescent Cu NCs@*p*-MBA with self-assembly induced emission (SAIE) properties were synthesized via a facile and rapid one-pot method. DFT analysis revealed the precise structure of the cyclic trinuclear Cu NCs@*p*-MBA and combined with MD simulation, the sensing mechanism of the assembled aggregates with high sensitivity in response to volatile ammonia species was explored. The simulation captured the changes in the supramolecular driving forces (including electrostatic interactions, van der Waals forces, and hydrogen bonds) within nanoaggregates during analyte detection, providing further information on the dynamic behaviors and interactions between Cu NCs@*p*-MBA and ammonia at the atomic scale, which revealed the nanoscale kinetic mechanism of structural transformation of Cu NCs@*p*-MBA and quenching of fluorescence. Cu NCs@*p*-MBA with SAIE properties held the advantages in building solid-state sensors, and the developed fluorescent labels exhibit the potential for applications in the nondestructive and in situ monitoring of seafood freshness.

## Supporting Information Available

SEM images, size distributions, AFM images, SEM-EDX, and element analysis of the self-assembled Cu NCs@*p*-MBA nanosheets; PL spectra and PL lifetime spectra of Cu NCs@*p*-MBA using different ratios of Cu to *p*-MBA; TEM images and PL spectra of the self-assembled Cu NCs@*p*-MBA nanosheets before and after storage; fluorescence photographs of the sensing labels before and after storage (PDF)
