# Supplementary material for: Assembly-Induced Emission of Copper Nanoclusters: Revealing the Sensing Mechanism for Detection of Volatile Basic Nitrogen in Seafood Freshness On-Site Monitoring
Source: ACS Appl Mater Interfaces. 2024 Jan 23;16(5):6533–47. doi: 10.1021/acsami.3c13321 (PMC10859926; doi:10.1021/acsami.3c13321)
Supplement: Supplementary file 1 — am3c13321_si_001.pdf [file am3c13321_si_001.pdf]

Supporting Information

Assembly-Induced Emission of Copper Nanoclusters: Revealing the Sensing Mechanism for Detection of Volatile Basic Nitrogen in Seafood Freshness On-Site Monitoring

Chenyue Zhou [a, b, c], Da-Wen Sun [a, b, c, d,*], Ji Ma [a, b, c, e, *], Anjun Qin [e], Ben Zhong Tang [e, f], Xiao-Ru Lin [g], Shi-Lin Cao [g]

[a] School of Food Science and Engineering, South China University of Technology, Guangzhou 510641, China

[b] Academy of Contemporary Food Engineering, South China University of Technology, Guangzhou Higher Education Mega Centre, Guangzhou 510006, China

[c] Engineering and Technological Research Centre of Guangdong Province on Intelligent Sensing and Process Control of Cold Chain Foods, & Guangdong Province Engineering Laboratory for Intelligent Cold Chain Logistics Equipment for Agricultural Products, Guangzhou Higher Education Mega Centre, Guangzhou 510006, China

[d] Food Refrigeration and Computerized Food Technology (FRCFT), Agriculture and Food Science Centre, University College Dublin, National University of Ireland, Belfield, Dublin 4, Ireland

[e] State Key Laboratory of Luminescent Materials and Devices, Center for Aggregation-Induced Emission, South China University of Technology, Guangzhou 510640, China

[f] Shenzhen Institute of Aggregate Science and Technology, School of Science and Engineering, The Chinese University of Hong Kong, Shenzhen 518172, China

[g] Guangdong Key Laboratory of Food Intelligent Manufacturing, Foshan University, Foshan 528000, China

[*] Corresponding authors. E-mail: dawen.sun@ucd.ie; URLs: http://www.ucd.ie/refrig, http://www.ucd.ie/sun

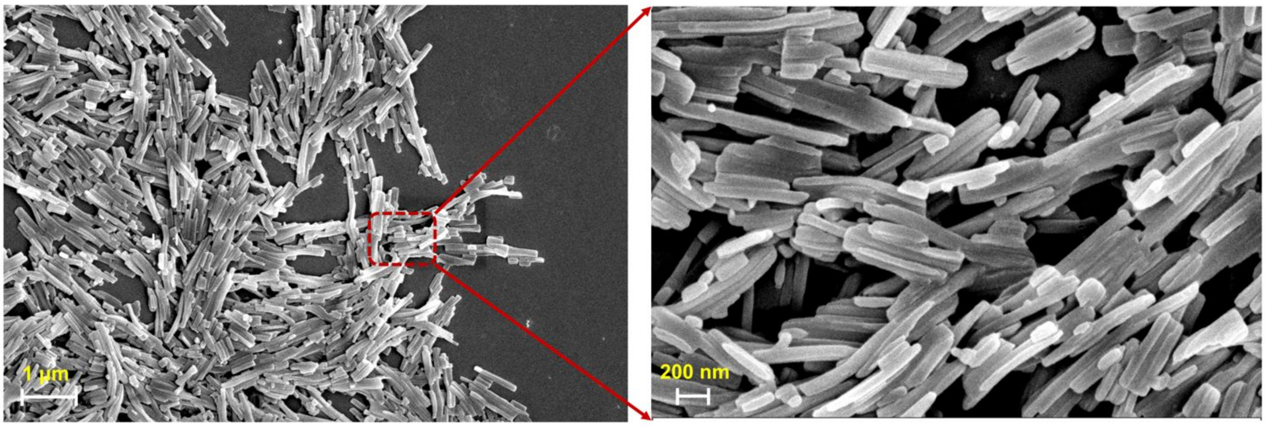

Figure S1. SEM images of the self-assembled Cu NCs@p-MBA nanosheets.

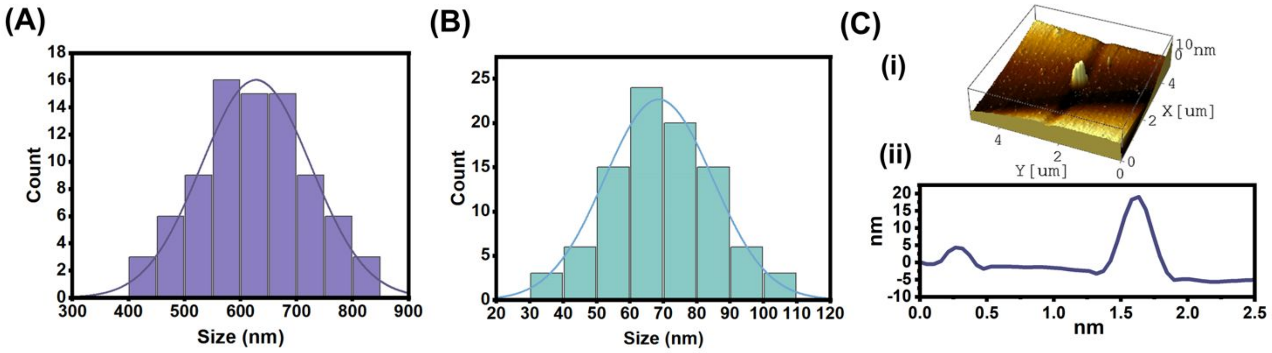

Figure S2. (A) Size histogram of the length of the nanosheets with a fitted curve. (B) Size histogram of the width of the nanosheets with a fitted curve. (C) AFM images of the self-assembled Cu NCs@p-MBA nanosheets, including (i) the topography image of the nanosheets and (ii) the height profile of the nanosheets.

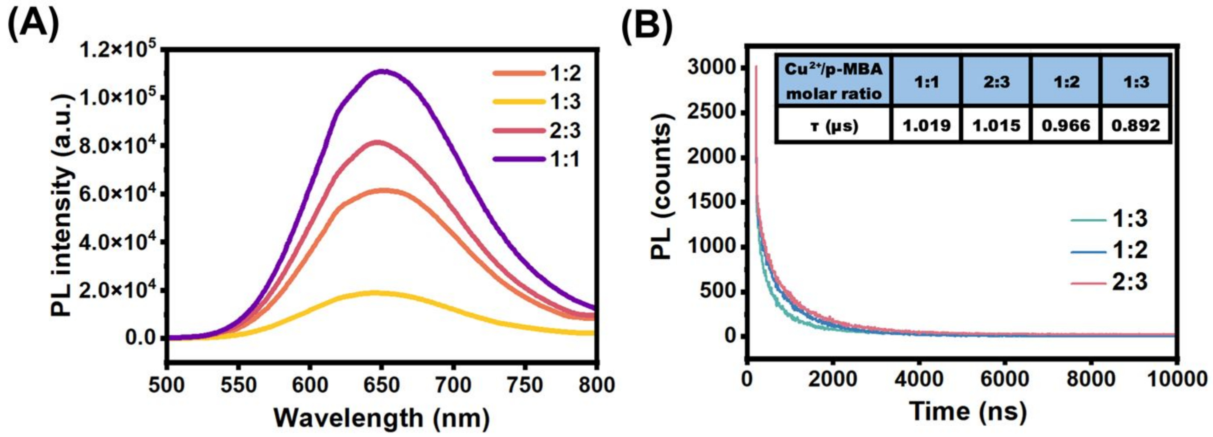

Figure S3. (A) The PL spectra of Cu NCs@p-MBA using Cu to p-MBA ratios of 1:1, 1:2, 2:3, and 1:3. (B) The PL lifetime spectra of Cu NCs@p-MBA using Cu to p-MBA ratios of 1:2, 2:3, and 1:3 (inset: the table of calculated PL lifetime of Cu NCs@p-MBA using Cu to p-MBA ratios of 1:1, 1:2, 2:3, and 1:3).

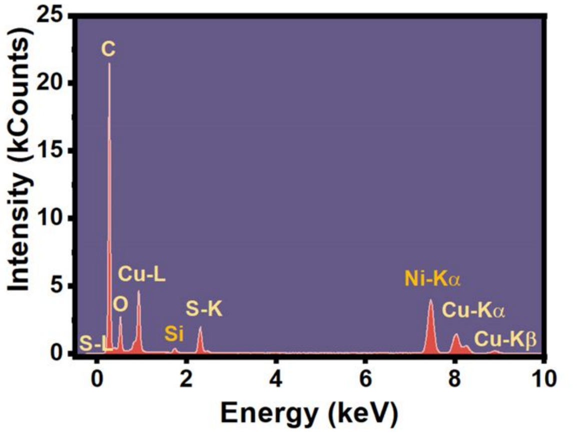

Element	C	O	S	Cu
Wt%	59.53	22.92	5.67	11.88
At%	72.92	21.78	2.59	2.71

Figure S4. SEM-EDX and element analysis of the self-assembled Cu NCs@p-MBA nanosheets.

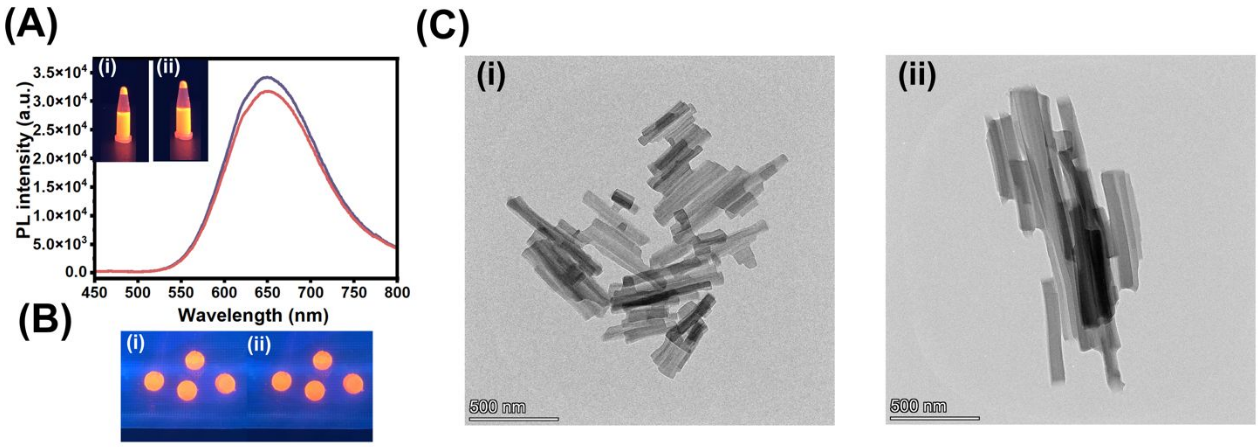

Figure S5. (A) The fluorescence emission spectra of freshly synthesized Cu NCs@p-MBA (purple line) and Cu NCs@p-MBA stored at 4 °C for 3 months (red line) (inset: the photograph of Cu NCs@p-MBA under UV light before (i) and after (ii) being stored). (B) The fluorescence photographs of the fabricated sensing labels loaded with Cu NCs@p-MBA before (i) and after (ii) being stored at 4 °C for 1 month. (C) TEM image of the self-assembled Cu NCs@p-MBA before (i) and after (ii) being stored at 4 °C for 3 months.
